# Glycoprotein Ib activation by thrombin stimulates the energy metabolism in human platelets

**DOI:** 10.1371/journal.pone.0182374

**Published:** 2017-08-17

**Authors:** Norma Corona de la Peña, Manuel Gutiérrez-Aguilar, Ileana Hernández-Reséndiz, Álvaro Marín-Hernández, Sara Rodríguez-Enríquez

**Affiliations:** 1 Unidad de Investigación en Trombosis, Hemostasia y Aterogénesis, Hospital Carlos McGregor, México City, México; 2 Center for Human Nutrition, Washington University School of Medicine, St. Louis, MO, United States of America; 3 Departamento de Bioquímica, Instituto Nacional de Cardiología, México City, México; 4 Laboratorio de Medicina Traslacional, Instituto Nacional de Cancerología, México City, México; Ludwig-Maximilians-Universitat Munchen, GERMANY

## Abstract

Thrombin-induced platelet activation requires substantial amounts of ATP. However, the specific contribution of each ATP-generating pathway *i*.*e*., oxidative phosphorylation (OxPhos) versus glycolysis and the biochemical mechanisms involved in the thrombin-induced activation of energy metabolism remain unclear. Here we report an integral analysis on the role of both energy pathways in human platelets activated by several agonists, and the signal transducing mechanisms associated with such activation. We found that thrombin, Trap-6, arachidonic acid, collagen, A23187, epinephrine and ADP significantly increased glycolytic flux (3–38 times *vs*. non-activated platelets) whereas ristocetin was ineffective. OxPhos (33 times) and mitochondrial transmembrane potential (88%) were increased only by thrombin. OxPhos was the main source of ATP in thrombin-activated platelets, whereas in platelets activated by any of the other agonists, glycolysis was the principal ATP supplier. In order to establish the biochemical mechanisms involved in the thrombin-induced OxPhos activation in platelets, several signaling pathways associated with mitochondrial activation were analyzed. Wortmannin and LY294002 (PI3K/Akt pathway inhibitors), ristocetin and heparin (GPIb inhibitors) as well as resveratrol, ATP (calcium-release inhibitors) and PP1 (Tyr-phosphorylation inhibitor) prevented the thrombin-induced platelet activation. These results suggest that thrombin activates OxPhos and glycolysis through GPIb-dependent signaling involving PI3K and Akt activation, calcium mobilization and protein phosphorylation.

## Introduction

After thrombin, ADP, collagen, tromboxane A2 or epinephrine trigger platelet activation, platelets are remodeled to acquire their canonical functions [1]. Platelet remodeling requires high loads of ATP and induces strong activation of energy pathways [2]. A few studies have focused on establishing the biochemical mechanisms associated with platelet energy metabolism activation, while identifying the principal ATP supplier (OxPhos or glycolysis) after agonist/platelet activation. Pioneer studies revealed that human platelets activated by thrombin (2–10 U/mL), collagen (40 μg/mL) or arachidonic acid (48 μM), showed a significant increase (2–5 times) in the total cellular oxygen consumption in comparison to non-activated platelets [3, 4]. However, it should be noted that human platelets exhibit some non-mitochondrial oxygen consumption (such as xanthine oxido reductase, lipoxygenase and cyclooxygenase) [3–5], which may mask the actual OxPhos flux. Therefore, cellular oxygen uptake should be performed in the presence of saturating oligomycin, an inhibitor of mitochondrial ATP synthase, in order to discard all non-mitochondrial oxygen- consuming reactions and unveil the mitochondrial oxygen consumption linked to ATP synthesis. In these studies, it was observed that platelet respiration was highly sensitive to oligomycin (up to 80%), indicating that mitochondrial respiration mainly comes from OxPhos [5–7]. On the other hand, platelet aggregation was highly sensitive to the combination of mitochondrial (antimycin A or cyanide) and glycolytic (2-deoxyglucose) inhibitors; individually both types of inhibitors did not inhibit aggregation [4]. These last results revealed that (i) ATP derived from both energy pathways is required for platelet activation [3], and (ii) under stress conditions where one of the ATP suppliers is impaired, platelets compensate by synthesizing ATP required to carry out their functions [6].

Thrombin stimulated-oxygen consumption correlates with a significant increase (from 0.1 to 1 μM) in the intracellular calcium *vs*. non-activated platelets [8]. Spikes in intracellular calcium may activate the mitochondrial matrix pyruvate and 2-oxoglutarate dehydrogenases, whose calcium activation constants (*Ka*) are close to 1 μM (reviewed by [9]). Thrombin activates platelets through the protease-activated receptors (PARs) and the glycoprotein Ib (GPIb/IX/V) complex. GPIb/IX/V signaling pathway mediates (i) PI3k/Akt activation and (ii) protein phosphorylation [10], whereas PARs signaling mediates the activation of phospholipase C isoform β (PLCβ), PI3- and RhoA/Rho kinases [11]. Both GPIb/IX/V and PARs also mediate increases in intracellular calcium in thrombin-activated platelets [8].

Previous studies have described that Akt over-expression accelerates mitochondrial oxygen consumption in injured proximal tubular cells. This also correlates with a substantial increase in the activity of several respiratory chain enzymes such as NADH dehydrogenase, (NAD1) and cytochrome c oxidase (COX), ΔΨm and ATP production [12]. In addition, Akt activation induces glucose consumption in gliobastoma cells [13]. Thus, although a role for the GPIb/IX/V and PARs signaling pathways on platelet OxPhos has not been established, these signaling pathways associated with OxPhos/glycolysis stimulation could be active during platelet activation. Here we studied the changes in OxPhos and glycolysis induced by platelet-aggregation agonists at different levels of complexity by assessing changes insignaling protein contents and energy metabolism fluxes. The results show that the GPIb/Akt signaling pathway stimulated by thrombin may up regulate energy metabolism in platelets.

## Materials and methods

### Blood collection

Blood samples were obtained from 20 non drug-treated and healthy donors. All procedures followed the health protocol approved by the Mexican Social Security Institute Bioethics Committee (http://www.imss.gob.mx/sites/all/statics/profesionalesSalud/investigacionSalud/normativaInst/2000-024-001.pdf). Blood was obtained by routine venipuncture from the antecubital vein and collected in 0.4% trisodium citrate buffer.

### Platelet-rich plasma (PRP) separation

PRP was obtained at room temperature as described elsewhere [14]. Blood was centrifuged at 250 x g for 10 min and the PRP was carefully separated. The pellet was then centrifuged at 1000 x g to obtain platelet poor plasma (PPP). Platelet count was determined in PRP by using a Coulter T-890 cytometer (Coulter Diagnostics, Miami, FL) and adjusted to 225,000 ± 25,000 platelets per μL using autologous platelet poor plasma(PPP).

### Washed platelets

For some experiments PRP was centrifuged at 1000 x g for 10 min at room temperature to obtain a pellet that was re-suspended in saline solution (0.9% w/v NaCl) to 2 x 10^5^ platelets/μL.

### Platelet aggregation

Platelet aggregation was determined in human PRP (2x10^5^ platelets/μL) by the turbidimetric method described elsewhere [14], using an 810-CA Chrono-log aggregometer (Chrono-log corporation, Havertown, PA), and Aggro-link computer interface (Chrono-log corporation, Havertown, PA). Platelet aggregation was quantified as the change in transmittance at 37°C under stirring in response to any agonist such as 10 μM ADP, 2 μg/mL collagen, 50 μM epinephrine, 1.5 mg/mL ristocetin, 0.5 U/mL thrombin, 50 μM Ca^2+^ionophore A23187, 0.5 mM arachidonic acid, or 22 μM thrombin receptor activating peptide 6 (Trap-6).

### Oxygen consumption

Oxygen consumption was determined polarographically [15] in PRP (2x10^5^ platelets/μl) at 37°C by using a Strathkelvin Mitocell Oxymeter MT200 (Strathkelvin Instruments, Scotland, UK) in the presence or absence of agonist. OxPhos was determined from the 5 μM oligomycin-sensitive respiration. The oxygen solubility in Mexico City at 2240 m altitude and 37°C is 380 ng atoms oxygen/mL (190 μM O_2_) [15].

### Lactate production

For lactate production, PRP (4x10^5^ platelets/μl) was incubated for 0 (t = 0) and 5 (t = 5) minutes at 37°C in the presence of 5 mM glucose and each agonist was added at the beginning of the incubation time. The samples were precipitated with perchloric acid (final concentration 3% v/v) and neutralized with 3 M KOH/0.1 M Tris. Neutralized samples were used for lactate determination by measuring NADH absorbance change at 340 nm [16]. For a rigorous glycolysis determination, flux was assayed in the presence of 25 mM 2-deoxyglucose (2DG) to discard lactate generated by glutamine (glutaminolysis) [17]. For rigorous glutaminolysis determination, the lactate produced in 2DG-treated cells was also assayed in the presence of 10 μM rotenone. ATP contribution from both energy pathways was calculated as [18].

### Mitochondrial membrane potential (ΔΨm)

The ΔΨm was determined in phosphate based-buffer, pH 7.4. Washed platelets (1 mg protein/mL) were incubated at 37°C with 0.25 μM rhodamine 6G [19] or 2 μM 5,5´,6,6-tetrachloro-1,1´,3,3´-tetraethyl-benzimidazolyl-carbocyanine iodide (JC-1) [20]. For rhodamine, fluorescence was registered in a RF 5301PC spectrofluorophotometer (Shimadzu Scientific Instruments, Maryland, USA) using 480 nm and 565 nm for excitation and emission wavelengths, respectively. Maximal fluorescence indicating total ΔΨm was attained for each experiment after adding 2.5 μM CCCP. For JC-1 assays, washed platelets (1x10^4^ platelets/μL) were analyzed in a FACS Scan flow cytometer (Beckton Dickinson, San Jose, CA) at 37°C, the excitation wavelength was 488 nm and the emission wavelengths were 535 nm for green (FL1) and 590 nm for red (FL2). Following 2.5 μM CCCP treatment the mitochondrial membrane potential of the cells was eliminated, as demonstrated by the increase of the monomer emission [20].

### Western blotting

Western blotting was performed as described previously [19]. Washed platelets (2–3X10^6^ cells) in 1% IGEPAL NP40 and 100 mM protease inhibitor mix (Calbiochem; San Diego, CA, USA) were precipitated and separated under reducing and denaturalizing conditions by 10% polyacrylamide gel electrophoresis. The proteins were blotted to polyvinylidene (PVDF) membranes (BioRad; Hercules, CA, USA) and Western blot analysis was performed by immunoblotting with the following antibodies: PI3K (sites Tyr 458 and Tyr 199), P-PI3K, Akt (site Thr 308), P-Akt, or β-actin at 1:1000 dilutions (all antibodies were from Santa Cruz; CA, USA). The hybridization bands were revealed with the corresponding secondary antibodies (1:2000 dilutions) conjugated with horseradish peroxidase (Santa Cruz) and the ECL-plus detection system (Amersham, Buckinghamshire, UK). Densitometric analyses were performed using the Image J Software (NIH; Bethesda, MD, USA) and double normalization (100% intensity) was done by using α-tubulin as loading control, calculating the percentage of phosphorylated protein as phosphorylated protein/tubulin ratio. The percentage level of each enzyme or transporter shown in results represents the mean ± SD of at least three independent experiments.

### Statistical analysis

Data points represent means ± SD of at least 3 different assayed preparations. Differences between means were evaluated using the Student’s t-test with p < 0.05 considered as the criterion of significance.

## Results

### Effect of platelet agonists on the oxidative phosphorylation of platelet-rich plasma

**Fig 1:**
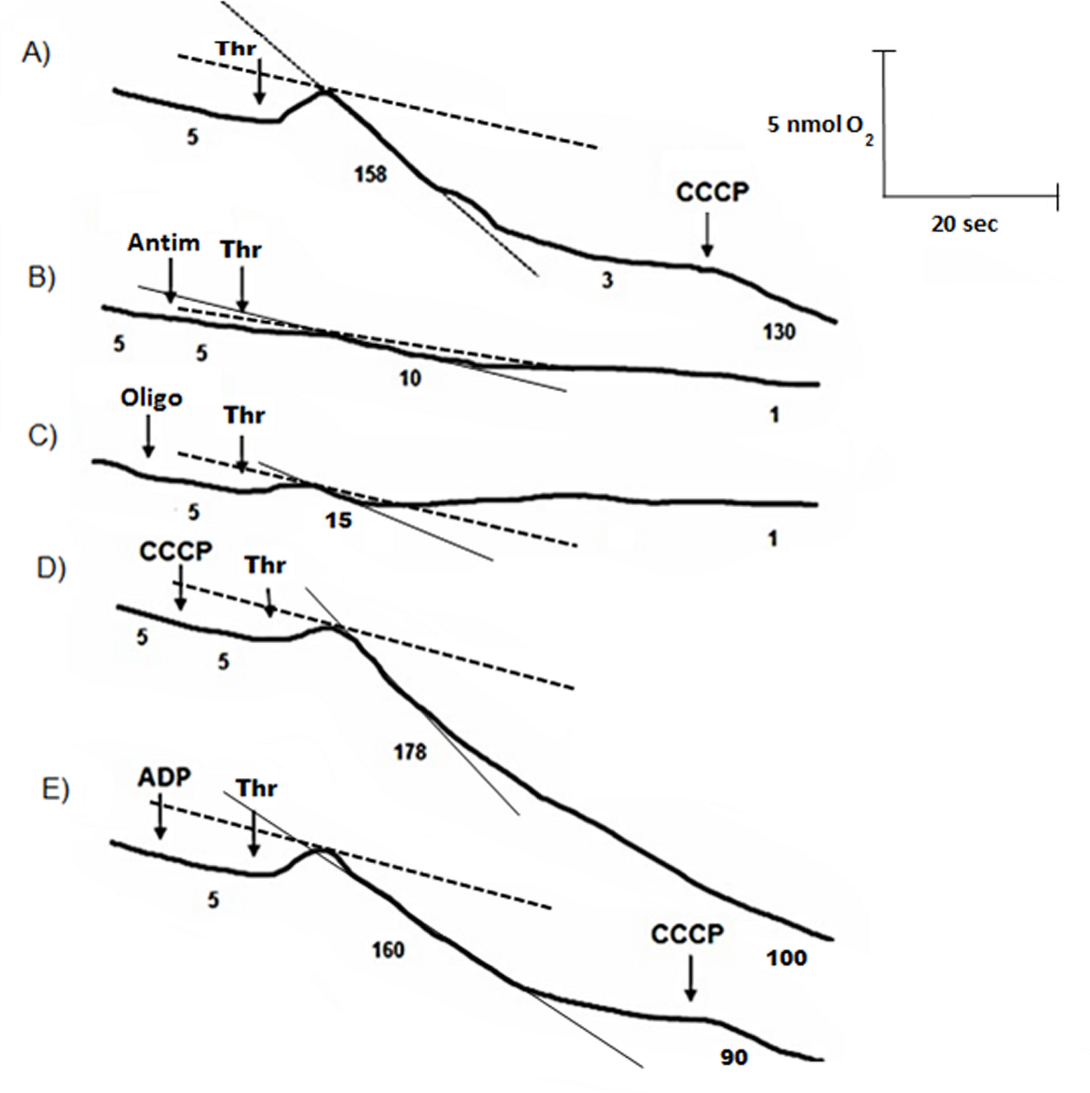
Effect of thrombin (Thr), OxPhos inhibitors and uncouplers in platelet oxygen consumption. Oxygen consumption in platelet-rich plasma was determined as described under “Material and Methods”. Arrows show Thr (0.5 U/ml), ADP (10 μM); CCCP (2.5 μM), oligomycin (Oligo, 5 μM) or antimycin (Antim, 5 μM) additions. Numbers under the traces indicate the rate of oxygen consumption in nanogram atom oxygen/min/10^9^ platelets. The lines over each trace indicate the change in oxygen consumption before (dashed lines) and after (solid lines) the addition of thrombin. Experimental traces are representative of at least 4 independent measurements.

Platelet oxygen consumption was evaluated in platelet-rich plasma (PRP), which preserves the platelets' physiological environment. In the absence of agonists, total oxygen consumption was abolished by oligomycin, indicating that in non-activated platelets oxygen consumption was fully linked to mitochondrial ATP supply (Table 1). Thrombin, arachidonic acid and collagen stimulated (2–33 times *vs*. non activated platelets) total oxygen consumption as well as OxPhos flux (Table 1). Thrombin also induced an increased ΔΨm, suggesting a direct effect on both the ΔΨm-producing OxPhos moiety (*i*.*e*. the respiratory chain) and the ΔΨm-consuming OxPhos moiety (*i*.*e*. ATP synthase, adenine nucleotide translocator) (Table 1, S1 Fig). This agonist induced a rapid transitory stimulation of respiration rate (Fig 1A), which was highly sensitive to antimycin (Fig 1B) and oligomycin (Fig 1C) and slightly stimulated (10–15%) by the uncoupler CCCP (Fig 1D). The addition of exogenous ADP (i.e., another platelet agonist) did not affect the stimulation of thrombin-induced OxPhos (Fig 1E).

**Table 1:** Energy metabolism in agonist-activated human platelets-rich plasma.

	No agonist	+ Thr	+ Trap-6	+ Arach	+ Coll	+ A23	+ Epi	+ ADP	+ Risto
**Total O**_**2**_ **consumption**	5±2	165±60	3±1	57±0.9	23±5	28±9	10±1	5±2	4±0.2
**OxPhos**	5±2	163±4*	3±1	36±2*	19±6*	10±5	2 ± 0.2	5±2	4±0.2
**Δψ**_**m**_ **(AFU)**	95±23	139±11*	46±11	96±15	68±23	10±4	100±5	42±10	94±25
**ATP contribution (%)**	67±15	88±10	15±6	66±12	22±6	10±3	22±5	25± 4	56±25
**Total lactate production**	7±6	58±9*	56±3*	62 ± 9*	205±50*	320±62*	25±7	41±3.5*	12±3
**Glycolysis**	6±8	54±6*	41±4*	47 ± 7*	173±40*	229± 57*	18±7	38±1*	8±5
**Glutaminolysis**	0.4 ± 0.5	3.6 ± 3	14 ± 0.5*	15 ± 2*	32 ± 10*	110 ± 22*	7 ± 0.5*	4 ± 1*	4 ± 2
**ATP contribution (%)**	32±12	12±6	85±20*	34±15	78 ± 20*	90±16*	78±12*	75±21*	44±12

Total oxygen consumption and OxPhos are expressed in nanogram atom oxygen/min/10^9^ platelets. OxPhos was oligomycin-sensitive oxygen consumption. Mitochondrial membrane potential (ΔΨm) is expressed in arbitrary fluorescence units (AFU). otal lactate production, glycolysis and glutaminolysis are expressed in nmol lactate/min/10^9^ platelets. Glycolysis was calculated as the lactate production inhibited by 25 mM 2DG. Glutaminolysis was calculated as the lactate production resistant to 25 mM 2DG. The 2DG-resistant lactate was >90%sensitive to 10 μM rotenone. For ATP supply calculations, the rate of OXPhos was multiplied by a P/O ratio of 2.5; for glycolysis, it was assumed that 1 nmol ATP is produced *per* nmol formed lactate [15]. The data shown are the mean ± SD of at least 3 independent preparations. Abbreviations: Thr, 0.5 U/mL thrombine; 22 μM Trap-6; Arach, 0.5 mM arachidonic acid; Coll, 2 μg/mL collagen; A23, 50 μM A23187; Epi, 50 μM epinephrine; 10 μM ADP; Risto, 1.5 mg/mL ristocetin.

*P< 0.05 vs. non agonist-activated platelets.

In contrast, OxPhos stimulation induced by collagen or arachidonic acid did not correlate with an increased ΔΨm; in fact, ΔΨm was not affected by arachidonic acid at all. ΔΨm was depressed by collagen (or the Ca^2+^ ionophore A23187) in comparison to non-stimulated platelets (Table 1, S1 Fig). This last observation suggests that collagen and arachidonic acid preferentially stimulates the ΔΨm-consuming OxPhos moiety.

Epinephrine increased total respiration (2-times) but significantly depressed OxPhos (Table 1), whileTrap-6 and ristocetin had no significant role on OxPhos (Table 1). Trap-6 significantly decreased ΔΨm (Table 1, S1A Fig). Finally, ADP had no measurable impact on platelet oxygen consumption.

### Effect of platelet agonists on the lactate production of platelet-rich plasma

All agonists assayed, except for ristocetin, increased the total lactate production (6–45 times) as well as the glycolytic rate (*i*.*e*., 2DG sensitive-lactate production) by 3–38 times (Table 1). Similarly, all agonists assayed including thrombin and ristocetin significantly increased glutaminolysis rate (*i*.*e*., 2DG resistant-lactate production) (Table 1).

### Contribution to ATP supply byOxPhos and glycolysis in activated platelet-rich plasma

OxPhos was the principal ATP-supplier in platelets activated with thrombin, arachidonic acid and ristocetin as well as in non-activated platelets (Table 1). In contrast, the main ATP-supplier in Trap-6-, collagen-, A23187-, epinephrine- and ADP-stimulated platelets was glycolysis (Table 1).

### Effect of glycolytic and OxPhos inhibitors on platelet function

Glycolytic and OxPhos inhibitors were added to platelet-rich plasma to assess the dependency of platelet aggregation on both energy sources. Platelet aggregation was inhibited by 2DG only in the presence of epinephrine (Table 2). Similar results were obtained for the OxPhos inhibitors antimycin A and oligomycin (Fig 1C and 1D). However, the combined use of 2DG and OxPhos inhibitors drastically diminished platelet aggregation induced by all agonists, except for ristocetin and A23187 (Table 2). On the contrary, energy inhibitors did not affect ristocetin-induced platelet aggregation. This suggests that aggregation induced by ristocetin may involve mechanisms not dependent on ATP as occurs with the other agonists. These results also indicate that there was not a differential sensitivity of platelet aggregation induced by the different agonists to either glycolysis or OxPhos inhibitors.

**Table 2:** Effect ofglycolytic and OxPhos inhibitors on agonist-induced platelet aggregation.

Agonist	Total aggregation	2DG	Antim	2DG/Antim	2DG/Oligo
**Thr**	90±10	88±9	89±8	25±8*	3±1*
**AA**	84±6	54±3	79±6	8±1*	8±3*
**Coll**	66±7	52±6	62±5	2±2*	18±6*
**A23**	75±7	40±5	75±4	15±3*	80±10
**Epi**	79±5	18±6*	79±4	7±0.8*	20±6*
**ADP**	60±8	40±4	46±26	2±2*	15±5*
**Risto**	84±6	70±8	80±6	80±1	83±7

Total aggregation is expressed in percentage of transmittance. Data shown are the mean ± SD of at least 3 independent preparations. Abbreviations are as in Table 1. 2-deoxyglucose, 25 mM 2DG; oligomycin, 5 μM Oligo; Antimycin, 5 μM Antim.

*P<0.05 vs agonists-activated platelets in the absence of inhibitor.

### Effect of GPIb inhibition on thrombin-stimulated OxPhos and glycolysis

**Fig 2:**
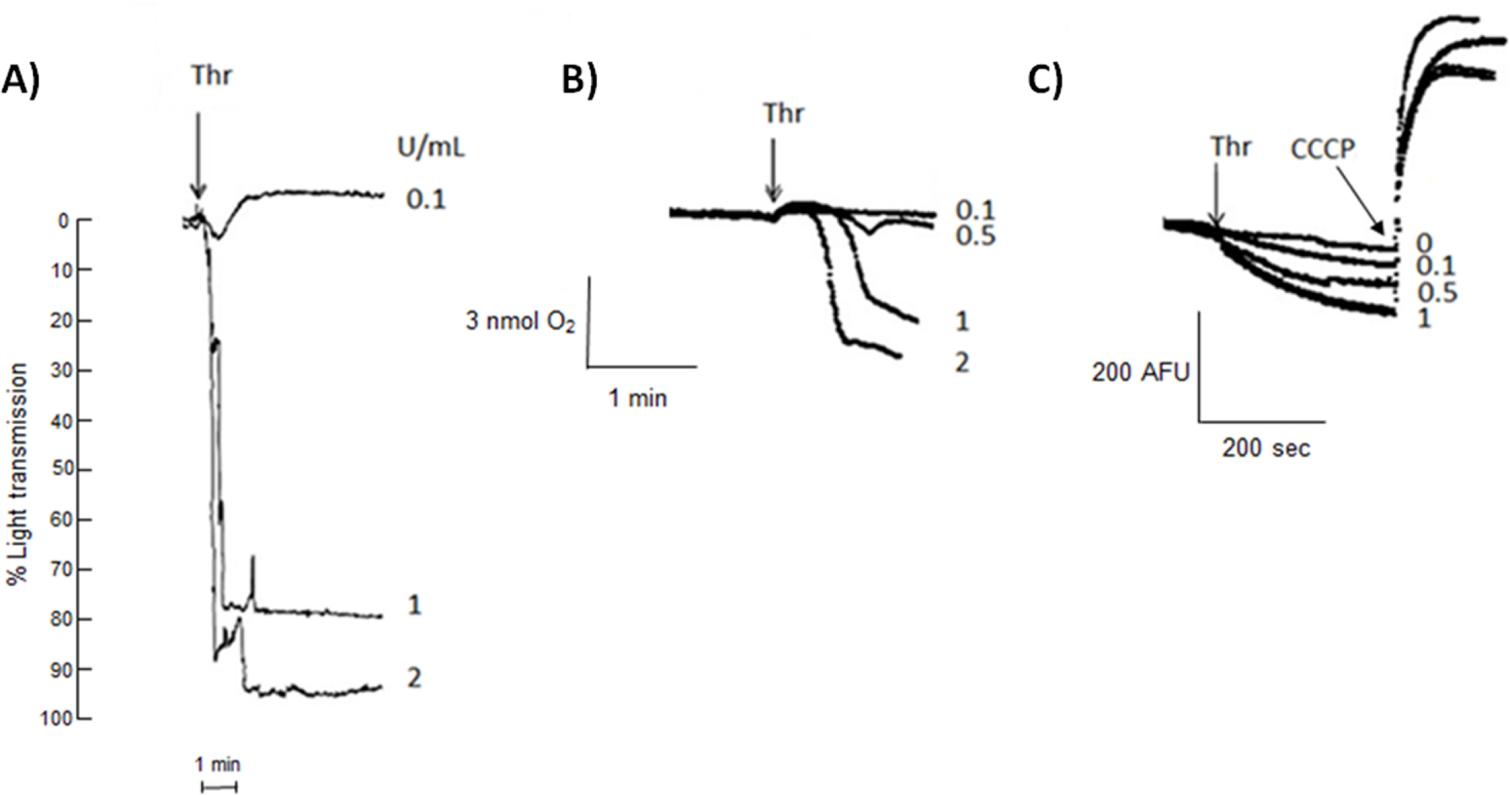
Effect of thrombin (Thr) in platelet aggregation and mitochondrial function. (A) Platelet aggregation; (B) platelet oxygen consumption; (C) mitochondrial membrane potentialin the presence of increasing concentrations of thrombin (Thr) as described in “Material and Methods” section. CCCP was added at 2.5 μM. AFU, arbitrary fluorescence units.

Thrombin induced platelet aggregation (Fig 2A) and increased total cellular respiration, OxPhos and ΔΨm (Table 1, Fig 2B and 2C) were achieved at similar doses (1–2 U/mL) suggesting a mechanistic link.

**Fig 3:**
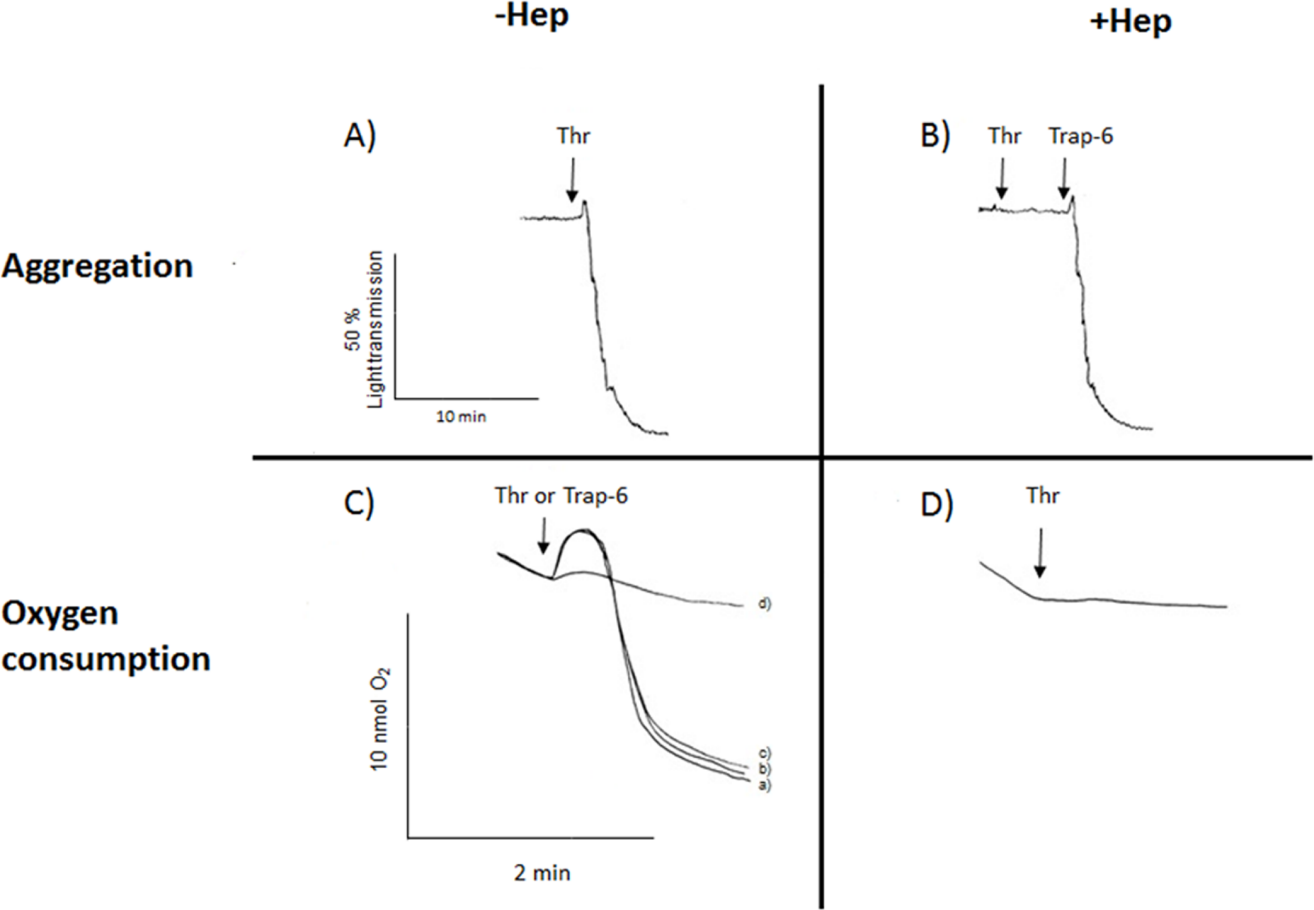
Effect of PAR-1 activation or GPIb inhibition on OxPhos stimulation induced by thrombin. Platelet aggregation (A,B) and oxygen consumption (C,D) were measured in thrombin (Thr) or Trap-6 stimulated platelets. Platelet rich plasma was incubated for 3 min with 1.5 mg/mL heparin (Hep, B,D) at 37°C under constant stirring. Afterwards, 0.5 U/mL thrombin or 22 μM Trap-6 was added as indicated by arrows. In (C), thrombin (a-c) was added in the presence of tirofiban (70 mg/ml) (b) or aspirin (1 μM) (c). In (d) only Trap-6 was added.

In order to determine the identity of the thrombin-activated receptors involved in the OxPhos activation, we examined the effects of Trap-6 that specifically activates PAR-1 [21] and heparin, which specifically inhibits GPIb-thrombin binding [21] on aggregation and oxygen uptake (Fig 3).

A typical human platelet aggregation profile in the presence of exogenous thrombin (0.5 U/mL) [4] is shown in Fig 3A. As expected, inhibition of GPIb receptor by heparin (Fig 3B) blocked platelet aggregation induced by thrombin. The addition of the PAR-1 activator Trap-6 restored platelet aggregation (Fig 3B). In parallel, the thrombin-induced increase in oxygen consumption was totally blocked by heparin (Fig 3D), indicating the participation of GPIb in this process. It is noted that heparin inhibited the thrombin induced-OxPhos stimulation at concentrations (Table 3) similar to those used for blocking the GPIb-thrombin binding receptor [22].

**Table 3:** Inhibition of thrombin-stimulated OxPhos (IC_50_) by GPIb ligands.

Inhibitor	IC_50_
**GPIb-thrombin binding antagonists**	
Heparin	5±1 μU/mL
Ristocetin	0.6±0.01 mg/mL
**PI3K inhibitors**	
Wortmannin	5 ± 1 nM
LY294002	25 ± 7 nM
**Tyr kinases inhibitor**	
PP1	200 ± 25 nM
**Ca**^**2+**^ **movement antagonists**	
ATP	2.5 ± 1 mM
Resveratrol	500 ± 70 nM
**Ca**^**2+**^ **movement inducer**	
Thapsigargin	2.5 ± 1 nM
**[cAMP] increase inducer**	
PGE1	0.4 ± 0.05 mM

OxPhos was determined as described under “Materials and Methods”. The inhibitors were assayed in the following concentration ranges: Heparin (0.01–0.4 mU/mL), ristocetin (0.5–3.5 mg/mL), wortmannin (0.5–10 nM), Ly294002 (2–100 nM), PP1 (0.1–1.5 mM), ATP (1–25 mM), resveratrol (0.5–20 μM), EGTA (0.5–45 mM), thapsigargin (0.5–6 nM) and PGE1 (0.25–5 mM). These inhibitors were incubated with PRP for 1 min prior to the addition of 0.5 U/mL thrombin. The data shown represent the mean ± SD of 3 different assayed preparations.

The binding of fibrinogen to the GPIIb/IIIa receptor and the formation of thromboxane are involved in platelet aggregation induced by thrombin [1]. To establish whether the activation of these mechanisms also affects mitochondrial function, inhibitors of GPIIb/IIIa (tirofiban) and tromboxane formation (aspirin) were assayed on thrombin-stimulated oxygen consumption (Fig 3C). Thrombin-stimulated oxygen consumption was not affected by tirofiban (Fig 3Cb) or aspirin (Fig 3Cc), indicating that both mechanisms are not implicated in the mitochondrial function stimulation. On the other hand, in platelets stimulated byTrap-6, no changes in the oxygen consumption were observed (Fig 3Cd), suggesting that PAR-1 was not implicated in the oxygen consumption stimulation.

Ristocetin activates von Willebrand factor, which in turn binds to GPIb on the thrombin binding site [23], thus decreasing thrombin binding to GPIb. Ristocetin also inhibited the activation of OxPhos induced by thrombin (Table 3), indicating that GPIb-coupled signaling pathway is involved in the thrombin-induced OxPhos activation. In addition, heparin (0.15 mU/mL) and ristocetin (1.5 mg/mL) significantly decreased (>95%) thrombin-stimulated glycolysis, again indicating that GPIb activation was involved in the thrombin-regulation of both energy pathways.

### Effect of modulators of PI3K/Akt, tyrosine-kinase, cAMP, ADP and calcium-dependent signaling pathways on the thrombin-stimulated OxPhos

It has been suggested that PI3K/Aktand calcium-dependent signaling pathways are involved in thrombin mediated GPIb activation [10]. In order to further demonstrate that PI3K/Akt and calcium-dependent signaling pathways are indeed involved in the stimulation of energy metabolism induced by thrombin, we tested the effects of inhibitors of PI3K (wortmannin and LY2940029) and scr-tyrosine-kinase (PP1, 4-amino-5-4-chlorophenyl-7-t-butyl pyrazolo-D-3, 4-pyridine) as well as the platelet adenylate cyclase activator (prostaglandin E1, PGE1) and inhibitors of (i) endoplasmic reticulum calcium-release (resveratrol, ATP) and (ii) calcium ATPase (thapsigargin).

Wortmannin and LY2940029 at nanomolar doses and PGE1 at high doses (>0.5 mM), compared to those required to block the GPIb-dependent platelet binding to thrombin [10], inhibited the thrombin-induced OxPhos activation (Table 3). This suggests that PI3K/Akt and adenylate cyclase are involved in the thrombin-induced OxPhos activation. Since respiration was only slightly affected (20%) by high PP1 (4 μM), scr-tyrosine-kinase appeared to have a minor role on thrombin-stimulated OxPhos (Table 3). In turn, resveratrol and ATP inhibited, while thapsigargin increased, the thrombin stimulated OxPhos at doses similar to those able to modulate intracellular calcium [24–26], suggesting that OxPhos activation by thrombin is mediated by calcium fluctuations.

Other mechanisms related to the activation of GPIb involve some nucleotides such as ADP or cAMP. It has been shown that the the GPIb-mediated aggregation induced by thrombin is strongly inhibited by apyrase which hydrolyses platelet-secreted ADP [10]. However, we observed that incubation with apyrase (0.5 U/mL) did not affect thrombin-OxPhos stimulation (data not shown).

### PI3K and Akt phosphorylation in response to thrombin

**Fig 4:**
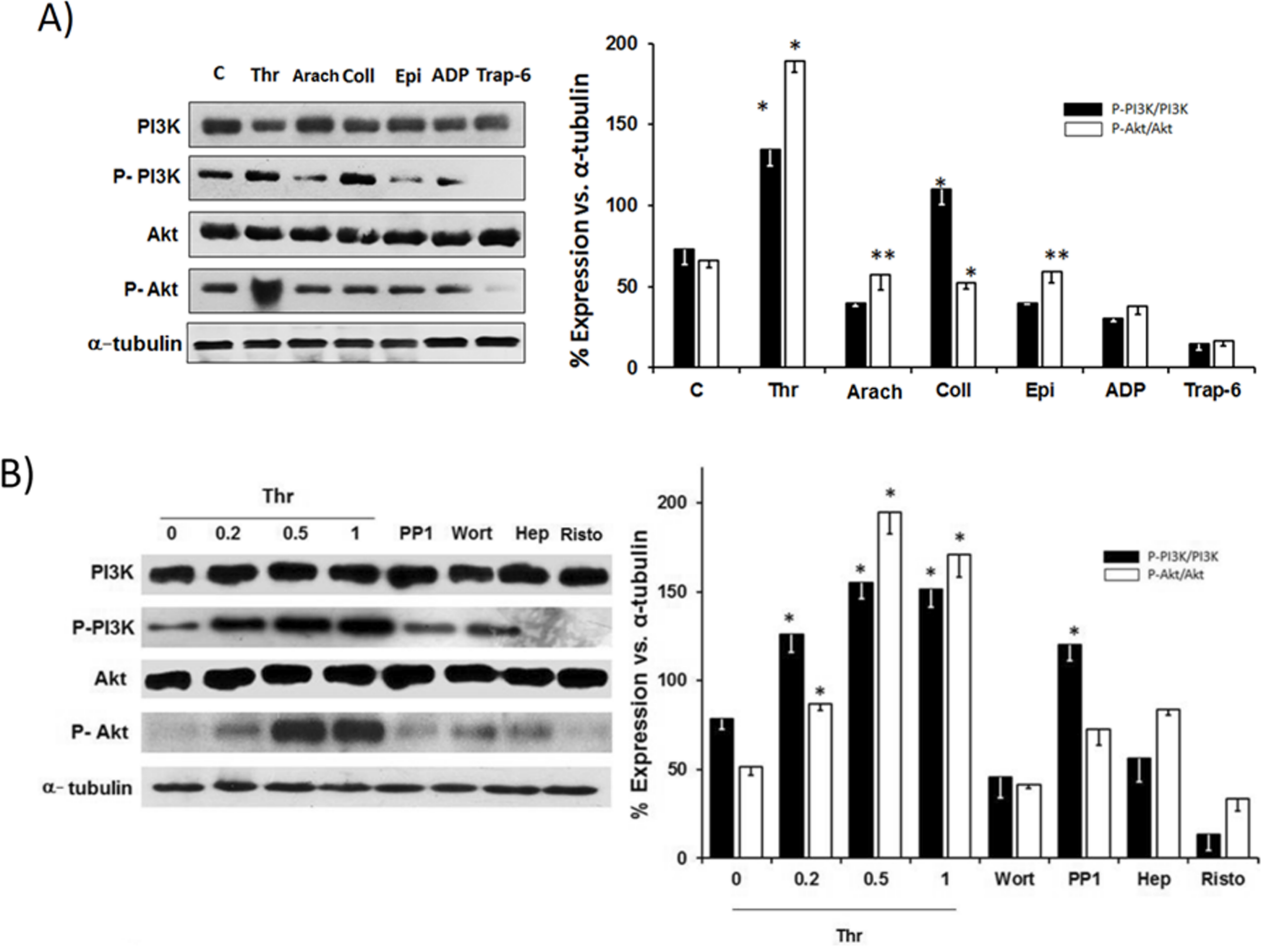
Total and phosphorylated PI3K and Akt contents in thrombin and other agonist-activated platelets. Non-phosphorylated and phosphorylated PI3K and Akt were detected with specific antibodies as described under “Material and Methods”. (A) PRP was incubated at 37°C for 5 min under stirring in the presence of thrombin, Thr (0.5 U/mL); arachidonic acid, Arach (0.5 mM), collagen, Coll (2 μg/ml), epinephrine, Epi (50 μM), ADP (10 μM), or Trap-6 (22 μM). (B) PRP was incubated in the presence of the indicated concentrations of thrombin (U/mL, lane 1–4), or thrombin (0.5 U/ml) and PP1 0.5 μM (lane 5), wortmannin 20 nM (lane 6), heparin 0.1 mU/mL (lane 7) or ristocetin 1.5 mg/mL (lane 8). Data represent mean ± SD of at least 4 independent determinations. *P<0.05 vs. control (non-activated platelets); **P<0.01 vs. control.

To further establish the role of PI3K/Akt in thrombin induced OxPhos stimulation, changes in the total and active (phosphorylated) isoforms of PI3K and Akt were evaluated after thrombin treatment (Fig 4). Indeed, thrombin (2–3 times) promoted a significant increase in the phosphorylated PI3K (p-PI3K) and AKT (p-AKT) contents, whereas arachidonic acid, epinephrine, ADP, collagen and Trap-6 were ineffective. Collagen caused a significant PI3K phosphorylation (50% vs. non-activated platelets) but Akt phosphorylation was negligible (Fig 4A). The dose-dependent increase in PI3K and Akt phosphorylation induced by thrombin was blocked by PP1, wortmannin, heparin and ristocertin (Fig 4B) indicating that the PI3K/Akt pathway was certainly involved in the GPIb-dependent thrombin-induced OxPhos stimulation and platelet aggregation according to Adam et al., [10].

## Discussion

Platelet activation is a highly energy demanding cellular process [2]. In consequence, platelet aggregation agonists increase energy metabolism by different mechanisms [3–5]. Thrombin particularly thrombin caused a strong stimulation of both glycolysis and OxPhos, being OxPhos the principal ATP supplier; whereas arachidonic acid and collagen also stimulated OxPhos but to a much lesser extent. On the contrary, collagen promoted a stronger stimulation of glycolysis compared to thrombin- or arachidonic acid- stimulated platelets. Therefore, glycolysis predominated in the supply of ATP.

Previous studies show that treatment with thrombin, arachidonic acid and collagen results in increased cytosolic and mitochondrial calcium levels [8, 27, 28] at concentrations where several OxPhos enzymes (FAD-glycerol phosphate dehydrogenase, pyruvate dehydrogenase, NAD^+^-isocitrate dehydrogenase and α-ketoglutarate dehydrogenase) are fully activated (0.1–1 μM), contributing to the generalized OxPhos activation observed after agonist treatment [8]. Activation of OxPhos flux in thrombin-stimulated platelets correlated with an enhanced Δψm further supporting the proposal that thrombin activates Krebs cycle enzymes for NADH production-increasing the electron transfer for proton pumping in the respiratory chain. Contrariwise, increased calcium activates the translocation of glucose transporter type 3 (GLUT3) to the plasma membrane [29]. Elevation of functional glucose transporters is associated with a significant increase in the glycolytic flux because GLUTs represent one of the main glycolytic controlling steps [30]. Ca^2+^ may also stimulate phosphofructokinase type I (PFK-I) activity through the phosphofructokinase type 2 (PFK-2) activation, the enzyme involved in the synthesis of fructose 2,6 bisphosphate, a potent PFK-I activator [31]; and Ca^2+^ -mediated activation of glycogen phosphorylase for glycogen breakdown and glucose-6-phosphate synthesis [32]. Accordingly, here we found that thrombin and other agonists increase the glycolytic flux.

Activation of OxPhos also correlated with an increased glutaminolysis rate, as previously reported [6, 17]. Glutamine oxidation requires of the activation of several mitochondrial enzymes (glutaminase, glutamate dehydrogenase, alanine-asparte transaminase) as well as an active Krebs cycle and an accelerated efflux of mitochondrial malate to cytosol for its onward transformation to pyruvate and oxidation to lactate [6, 33]. Perhaps agonists activate enzymes involved in glutaminolysis or in other mitochondrial intermediary supplier-pathways (β-oxidation, ketone bodies and amino acid degradation, etc).

Ravi et al. [6] also reported that thrombin stimulated OxPhos in human platelets but to a lesser extent (1.5-times) than that found in our study (33-times). This difference may be attributed to the non-fresh human platelets used by Ravi et al., *i*.*e*. platelets were isolated after 6–8 days blood collection, which in turn drastically impairs mitochondrial function [34]. For this reason, the present study was carried out using fresh platelets (PRP). In addition, the glycolysis rate in our thrombin-stimulated fresh platelets was lower than that reported by Ravi et al. [6], in agreement with reports indicating that old platelets exhibit high lactate production [35].

### Mitochondria is the principal ATP supplier in thrombin-induced platelet activation

Both glycolytic and OxPhos fluxes were significantly stimulated by several platelet agonists. Compared with non-stimulated platelets where both pathways equally contributed to ATP supply (50%), thrombin promoted a shift in the energy dependence under which mitochondria provided more than 85% of the total ATP. Contrariwise, Trap-6, collagen, A23187, epinephrine and ADP promoted that glycolysis was the principal ATP supplier. This takes relevance because independently of agonist used, the activated platelets always will have a constant energy supply. Therefore, the combined use of glycolytic and OxPhos inhibitors may promote a drastic abolishment of the majority of platelet aggregation inducers. Akkerman and Holmsen [4] also found that platelet aggregation is highly sensitive to combined OxPhos plus glycolytic inhibitors. They concluded that OxPhos and glycolysis may compensate for each other in order to provide ATP for aggregation. However, our experiments show that both energy pathways supply the ATP required for platelets aggregation after collagen, epinephrine and arachidonic acid treatment.

### Thrombin activates OxPhos and glycolysis through GPIb-dependent signaling

Although most of the agonists analyzed here promoted changes in the energy pathways, we mainly focused on thrombin signaling because it is the physiological agonist that causes the strongest response in platelets (reviewed in [1]). Thrombin promotes the activation of multiple membrane receptors including GPIb/V/IX complex and some PARs [36] whose activation is linked to increases in total cellular respiration [4]. In this regard, thrombin signaling in platelets depends principally on PAR-1 and GPIb receptors [10]. Platelets also contain the thrombin receptor PAR-4 involved in the platelet activation [36], but under the experimental conditions used in this study (low thrombin concentrations and short incubation times), any role of PAR-4 is negligible [37].

Analysis with the PAR-1 activator Trap-6, at doses in which PAR-1 is significantly activated (from 60–100%) in human platelets [21] showed that Trap-6 did not modify the rates of OxPhos, suggesting that PAR-1 was not involved in the platelet energy metabolism activation induced by thrombin. In the presence of ristocetin or heparin -both inhibitors of GPIb [22, 23]- thrombin was not able to induce glycolysis or OxPhos activation. It should be noted that ristocetin and heparin at doses used in this study failed to affect any of the energy pathways (data not shown), which supports the conclusion that GPIb receptor is involved in the thrombin-induced stimulation of OxPhos and glycolysis. Moreover, a GPIb-independent effect of heparin on thrombin-induced activation of platelets seems unlikely, because heparin binds to thrombin-orGPIb-receptors using the same binding domain [22]. Furthermore, heparin inhibited thrombin-induced platelet aggregation but not Trap-6, indicating that PAR-1 activation was not impaired by heparin, as also reported by others [38]. Thus, the evidence suggests that heparin specifically inhibits only GP1b-dependent thrombin-induced responses.

The inhibitory effects of ristocetin and heparin on thrombin-induced platelet aggregation involves strong inhibitor/receptor interaction [22, 23], thus perhaps blocking the subsequent downstream intracellular calcium rise and hence the stimulation of glycolysis and OxPhos. Plasmin, a potent GPIb destabilizing agent, also promotes a strong depression in the oxygen consumption of thrombin-treated human platelets [39] clearly indicating a central role for GPIb in modulating mitochondrial function perhaps through intracellular calcium movements.

### Molecular mechanisms involved in the GPIb-mediated energy pathways activation by thrombin

GPIb may coordinate with other receptors such as the adenosine diphosphate receptor subtype 12 (P2Y12) for adequate signaling and further P2Y12 activation requires extracellular ADP interaction [10]. However, the incubation with the extracellular ADP scavenger apyrase did not affect the OxPhos and glycolysis stimulation induced by thrombin. This suggests that (a) secreted ADP does not play a role in this process or that (b) ADP secretion takes place downstream of thrombin-induced energy pathways stimulation.

In addition to GPIb, GPIIb/IIIa plays a pivotal role in platelet-platelet interaction induced by thrombin and other agonists [40]. To assess whether GPIIb/IIIa has a role in the thrombin-induced energy pathways stimulation, the antagonist tirofiban was tested. Tirofiban at doses reported to inhibit GPIIb/IIIa function (*i*.*e*. fibrinogen binding) [41] did not prevent the stimulation of OxPhos induced by thrombin, indicating that platelet aggregation through fibrinogen bridges is not a prerequisite for the stimulation of OxPhos induced by thrombin. Similarly, it became clear that thrombin-mediated thromboxane formation through cyclooxygenase activity was not involved in the energetic stimulation by thrombin, as aspirin did not perturb the thrombin-mediated stimulation of OxPhos. These results discarded a role for P2Y12 or GPIIb/IIIa in the thrombin activation of both energy pathways.

cAMP is a negative modulator of platelet aggregation induced by thrombin. Accordingly, PGE1 inhibited the stimulatory effect of thrombin on OxPhos, indicating that cAMP increase triggered by PGE1 has a negative control of thrombin-stimulated OxPhos. Accordingly, it has been demonstrated that cAMP decrease the ADP-mediated platelet activation through receptor P2Y12 promoting GPIbβ phosphorylation [42], which in turn may block platelet OxPhos.

### PI3K/Akt modulates energy metabolism in thrombin-activated platelets

GPIb/thrombin activates several signaling pathways such as PI3K/Akt, which increase the cytosolic Ca^2+^level and the phosphorylation (in the tyrosil residues) of several platelet proteins [10]. In assessing the hypothesis that the activation of energy pathways is mediated by GPIb/thrombin signaling, the phosphorylation status of PI3K and Akt was determined. As expected, thrombin promoted a strong phosphorylation (i.e., activation) of PI3K and Akt, as also reported by other groups [10]. Arachidonic acid, collagen, epinephrine and ADP also promoted the phosphorylation of PI3K and Aktbut to significantly lesser extent as reported in other studies [43]. The use of PI3K/Akt inhibitors prevented the thrombin mediated-stimulation of OxPhos at concentrations in which OxPhos and glycolysis are not affected [44]. This supports the explanation that the stimulation of energy metabolism is mediated by the PI3K/Akt signaling pathway.

Therefore, it can be concluded that there is a strong correlation between Akt activation and OxPhos and glycolysis stimulation in thrombin-activated platelets. In this regard, it has been considered that Akt may have regulatory roles on energy metabolism [45]. Insulin-like growth factor-1 and insulin promote the phosphorylation and translocation of Akt from the cytosol to the mitochondria [45].

Akt activation has been involved in the activation of mitochondrial or glycolytic function in several models. In isolated mitochondria of renal proximal tubular cells [12] the activation of Akt with a neurotoxic agent increased both citrate driven-state 3 mitochondrial respiration and Δψ_m_ by 38–50%, correlating with increased (39–55%) respiratory chain complex III and ATP synthase activities [12]. In murine pro-B lymphocytic, human osteosarcoma cells, Hepa1c1c7 cells, Akt activation was linked to an over-expression of glycolytic enzymes (GLUT1, hexokinase type 2) which in turn correlated with high glycolytic flux [13]. In bovine heart, Akt directly phosphorylates PFK2 leading to enhanced levels of fructose 2,6 bisphosphate, the most potent PFK-I activator, and thus increasing glycolysis [46].

### Activation of OxPhos by thrombin depends on calcium movements and platelet protein phosphorylation

ATP and resveratrol inhibit intracellular calcium mobilization in thrombin activated platelets [24–26]. Although it was demonstrated that resveratrol has multiple targets, at the concentrations assayed, it principally modifies the intracellular free calcium concentration after thrombine or collagen treatment [24, 47–49]. Since these two agonists partially prevented the stimulation of OxPhos by thrombin, it can be concluded that increased cytosolic Ca^2+^mediates the thrombin stimulation of OxPhos. At the doses assayed, resveratrol does not directly affect OxPhos [50].

## Concluding remarks

With these observations, we conclude that thrombin stimulates glycolysis and OxPhos through GPIb-triggered signaling involving phosphorylation of signaling proteins like Akt and perhaps calcium movements. Our results may contribute to the knowledge of the mechanisms involved in the regulation of energy metabolism in platelets and also to the elucidation of the mechanisms responsible for platelet metabolic regulation and function alterations especially in thrombosis-associated diseases [51].

## Supporting information

S1 Fig Mitochondrial membrane potential (ΔΨm) in agonist-activated platelets. (A) Washed platelets were incubated with rhodamine (0.25 μM) and 0.5 U/mL thrombin (Thr), 0.2 μg/ml collagen (Coll), 50 μM A23187 (A23) or 10 μM ADP. Signal was calibrated by the addition of 2.5 μM CCCP. ΔΨm is expressed in arbitrary fluorescence units (AFU). (B) For plasma rich platelets, cells were incubated with JC-1 (2 μM) and same agonist concentrations used in (A). Platelet regions were selected as CD42 positive events (data not shown). Representative images of at least 4 independent experiments.
(DOCX)
