# Supplementary material for: Glycoprotein Ib activation by thrombin stimulates the energy metabolism in human platelets
Source: PLoS One. 2017 Aug 17;12(8):e0182374. doi: 10.1371/journal.pone.0182374 (PMC5560607; doi:10.1371/journal.pone.0182374)
Supplement: S1 Fig — (A) Washed platelets were incubated with rhodamine (0.25 μM) and 0.5 U/mL thrombin (Thr), 0.2 μg/ml collagen (Coll), 50 μM A23187 (A23) or 10 μM ADP. Signal was calibrated by the addition of 2.5 μM CCCP. ΔΨm is expressed in arbitrary fluorescence units (AFU). (B) For plasma rich platelets, cells were incubated with JC-1 (2 μM) and same agonist concentrations used in (A). Platelet regions were selected as CD42 positive events (data not shown). Representative images of at least 4 independent experiments. (DOCX) [file pone.0182374.s001.docx]

SUPPLEMENTARY FIGURES

**S1 Figure Title: Mitochondrial membrane potential (ΔΨm) in agonist-activated platelets**


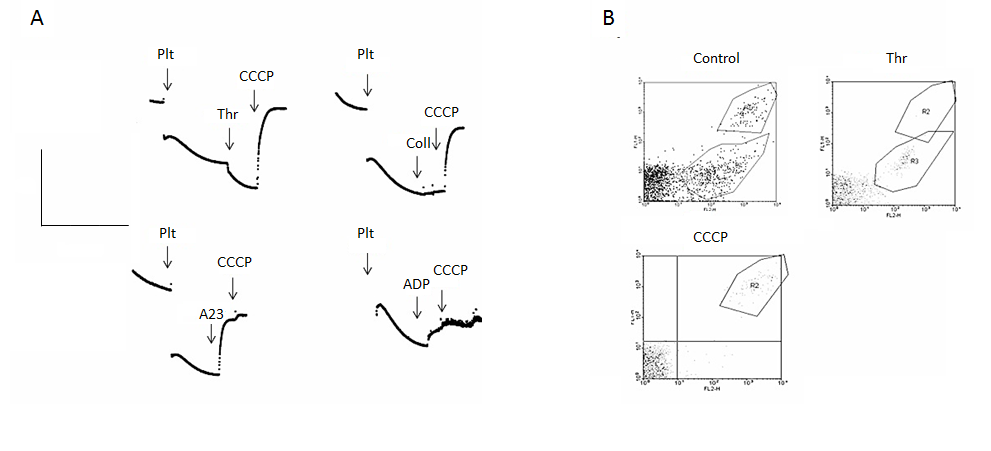


S1 Figure legend: (A) Washed platelets were incubated with rhodamine (0.25 µM) and 0.5 U/mL thrombin (Thr)), 0.2 µg/ml collagen (Coll), 50 µM A23187 (A23) or 10 µM ADP. Signal was calibrated by the addition of 2.5 µM CCCP. ΔΨm is expressed in arbitrary fluorescence units (AFU). (B) For plasma rich platelets, cells were incubated with JC-1 (2 µM) and same agonist concentrations used in (A). Platelet regions were selected as CD42 positive events (data not shown). Representative images of at least 4 independent experiments.
